# P-2074. Evaluation of Adherence and Access to Booster COVID-19 Vaccination in Persons with HIV

**DOI:** 10.1093/ofid/ofae631.2230

**Published:** 2025-01-29

**Authors:** Tara Krishna, Shobha Swaminathan

**Affiliations:** New Jersey Medical School, Newark, New Jersey; Rutgers New Jersey Medical School, Newark, New Jersey

## Abstract

**Background:**

COVID-19 disproportionately effects minority communities. Completion of the primary COVID-19 vaccination series (≥ 2 doses) is recommended for all American adults by the Centers for Disease Control and Prevention. A population with high mortality and morbidity related to COVID-19 is persons with HIV (PWH). The Infectious Diseases Practice (IDP) at University Hospital (UH) has been providing care to PWH for over 30 years, and it was one of the designated COVID-19 vaccination centers early in the pandemic. We compared rates of PWH who receive COVID-19 vaccine (incomplete: < 2, complete: ≥ 2) amongst different demographic factors to better understand subgroups that can benefit from additional messaging to promote vaccine uptake.

**Figure:**
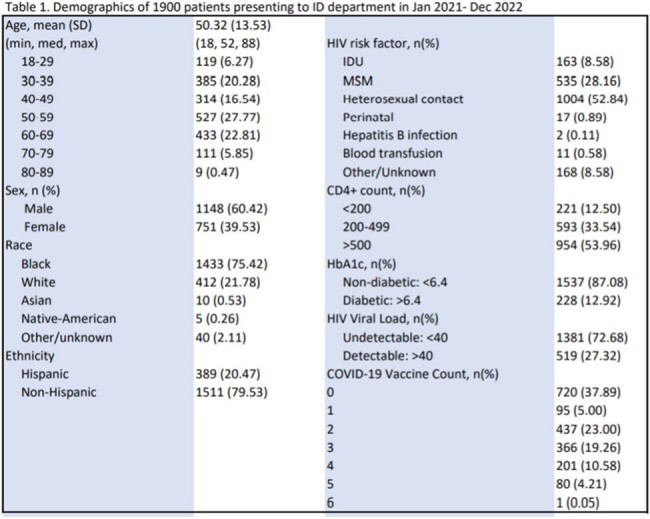

**Methods:**

A retrospective chart review study was conducted at the IDP at UH, Newark. Adult PWH (18-88 years old) who had an office visit to the clinic between January 2021-December 2022 were included. Demographic variables and COVID vaccine count were collected.

**Figure:**
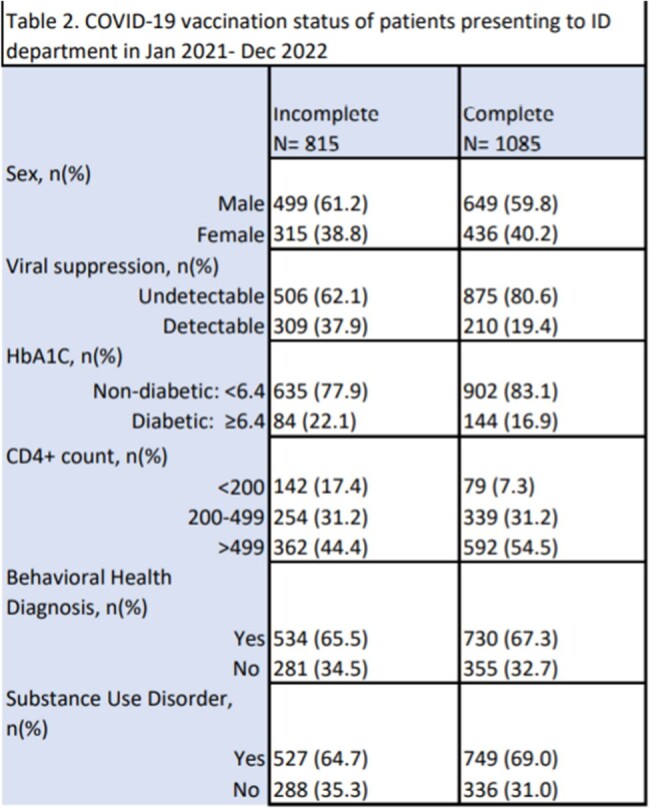

**Results:**

The IDP identified PWH (mean age 52, 75.4% Black, 20.4% Hispanic, 39.5% women) who had either complete (n=1085) or incomplete (n=815) primary vaccine schedules for COVID-19. The most common HIV risk factors were heterosexual contact (52.8%), homosexual contact (28.16%), and injection drug use (8.58%). 54% of patients had a CD4+ count > 499, 87.08% of patients had an HbA1c < 6.4, and 72.68% of patients had an HIV viral load that was undetectable.

Notably, PWH who completed the primary vaccination series were more likely to be undetectable (80.6%) years compared to PWH who did not complete their vaccination series (62.1%). Furthermore, PWH who completed the primary vaccination series were more likely to have an HbA1c< 6.4 (83.1%) compared to PWH who did not complete their vaccination series (77.9%). PWH who completed the primary vaccination series were more likely to have a CD4 count > 499 (54.4%) compared to PWH who did not complete their vaccination series (44.4%).

**Conclusion:**

Patients most at risk of not completing the primary COVID-19 vaccine schedule include PWH who are virally uncontrolled, persons with diabetes, or have a low CD4+ count. This indicates medical adherence should be targeted in such populations to improve their healthcare outcomes.

**Disclosures:**

Shobha Swaminathan, MD, Viiv Healthcare: Advisor/Consultant

